# Bidirectional mid-infrared communications between two identical macroscopic graphene fibres

**DOI:** 10.1038/s41467-020-20033-2

**Published:** 2020-12-11

**Authors:** Bo Fang, Srikrishna Chanakya Bodepudi, Feng Tian, Xinyu Liu, Dan Chang, Sichao Du, Jianhang Lv, Jie Zhong, Haiming Zhu, Huan Hu, Yang Xu, Zhen Xu, Weiwei Gao, Chao Gao

**Affiliations:** 1 MOE Key Laboratory of Macromolecular Synthesis and Functionalization, Department of Polymer Science and Engineering, Zhejiang University, 38 Zheda Road, 310027 Hangzhou, People’s Republic of China; 2 College of Micro-Nano Electronics, ZJU-Hangzhou Global Scientific and Technological Innovation Centre, State Key Laboratory of Silicon Materials and Modern Optical Instruments, Zhejiang University, 38 Zheda Road, 310027 Hangzhou, People’s Republic of China; 3 Zhejiang University/University of Illinois at Urbana-Champaign Joint Institute (ZJU-UIUC), Zhejiang University, 314400 Haining, Zhejiang People’s Republic of China; 4 Department of Chemistry, Zhejiang University, 310027 Hangzhou, Zhejiang People’s Republic of China

**Keywords:** Materials science, Nanoscience and technology, Optics and photonics

## Abstract

Among light-based free-space communication platforms, mid-infrared (MIR) light pertains to important applications in biomedical engineering, environmental monitoring, and remote sensing systems. Integrating MIR generation and reception in a network using two identical devices is vital for the miniaturization and simplification of MIR communications. However, conventional MIR emitters and receivers are not bidirectional due to intrinsic limitations of low performance and often require cryogenic cooling. Here, we demonstrate that macroscopic graphene fibres (GFs) assembled from weakly-coupled graphene layers allow room-temperature MIR detection and emission with megahertz modulation frequencies due to the persistence of photo-thermoelectric effect in millimeter-length and the ability to rapidly modulate gray-body radiation. Based on the dual-functionality of GFs, we set up a system that conducts bidirectional data transmission by switching modes between two identical GFs. The room-temperature operation of our systems and the potential to produce GFs on industrial textile-scale offer opportunities for simplified and wearable optical communications.

## Abstract (web summary)

Here, the authors design macroscopic multi-layered graphene fibres with light-emission and -detection dual functionality in the mid-infrared range, offering megahertz modulation frequencies and bidirectional data transmission operation.

## Introduction

Optical communications based on mid-infrared (MIR) light are vital in molecular “fingerprint” imaging, healthcare, and in a wide variety of applications including security, environmental monitoring, metrology, and atmospheric science^1–6^. Creating bidirectional MIR communication systems using two identical devices facilitates the design of simplified MIR-based optoelectronic systems. Such conceptual advances have been witnessed in visible and near-infrared regions, but not yet in the MIR range^7,8^. This is because the majority of traditional MIR detectors and emitters, e.g., narrow-band gap semiconductors^9,10^, are predominantly designed to be either emitters or detectors, and, therefore, are not compatible for dual-functionality with satisfactory performance.

Single and few-layer graphene (FLG) are a suitable choice for both MIR emission and detection. FLG can emit MIR through recombination of electrically injected electron-holes, or gray-body irradiation^11,12^. Besides, it can effectively detect MIR via direct band transition and due to the presence of longer hot-carrier lifetime^13,14^. However, despite their promises in terms of bandwidth and flexibility, their performance is still intrinsically limited by weak absorption and short light-matter interaction lengths in the MIR region. Microscale thick stacks of multilayer graphene can be considered as a viable alternative as it shows improved MIR absorption and emission due to increased interaction length. Yet, critical challenges, including a high recombination rate and short carrier transport lengths, remained. Recent reports suggest that the weak interlayer coupling can potentially minimize the out-of-plane scattering and exhibit intrinsic mobility like graphene, and effectively confine photoexcited carriers to individual layers^15,16^. Such uncorrelated multilayer graphene can retain hot electron relaxation times similar to single-layer graphene and sustain photo-thermoelectric effect for long spatial scales^16^. So far, there is no practical demonstration of room-temperature MIR communication in freestanding, flexible bulk graphene systems.

In this work, we use a scalable twist-draw protocol to assemble graphene nanosheets into macroscopic graphene fibers (GFs) with weakly coupled graphene layers. As a receiver, GF exhibits room-temperature MIR detection at 0.25 MHz as a result of photo-thermoelectric effect. As an emitter, GF is an efficient gray-body radiator to irradiate bias-dependent MIR spectrum at 10 MHz. Based on these merits, we developed the bidirectional MIR communication link using two identical GFs, where two GFs optically couple two separate electrical circuits and exchange information mediated by MIR light.

## Results

### Fabrication and characterization of GFs

**Fig. 1:**
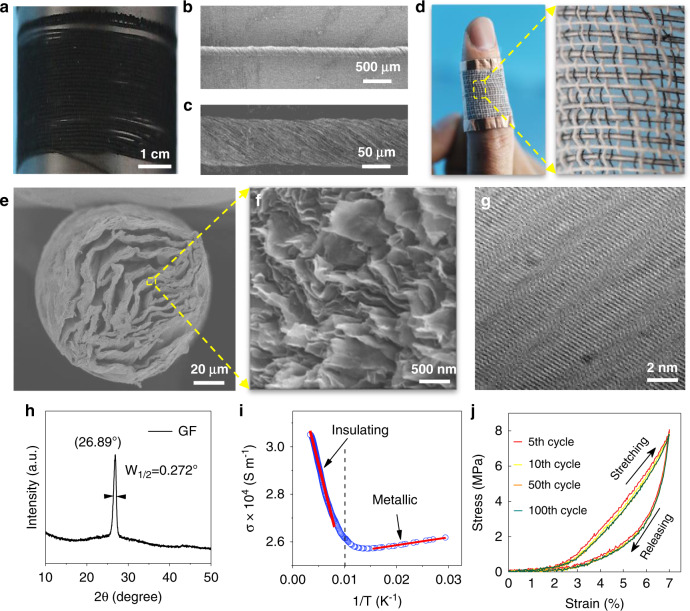
Structural characteristics of GFs. **a** Photograph of meters-long GF collected on a graphite roller. **b**, **c** SEM images of the GF surface at different magnifications. **d** Weaving continuous GFs into a cotton cloth and wearing the composite fabric on a finger. **e**, **f** SEM images of GF cross-section showing randomly stacked graphene sheets. **g** TEM image of a crystallized graphene sheet in GF. **h** XRD spectrum of GF. The full width at half maximum (*W*_1/2_) of ~0.272° indicates restoration of ~29.7 nm thick graphene crystals in GF as described by Scherrer equation (LC = 0.9*λ*/(*W*_1/2_ cos*θ*)). **i** Four-point electrical conductivity of GF as a function of inverse temperature. **j** Mechanical durability test of GF up to 100 cycles.

**Fig. 2:**
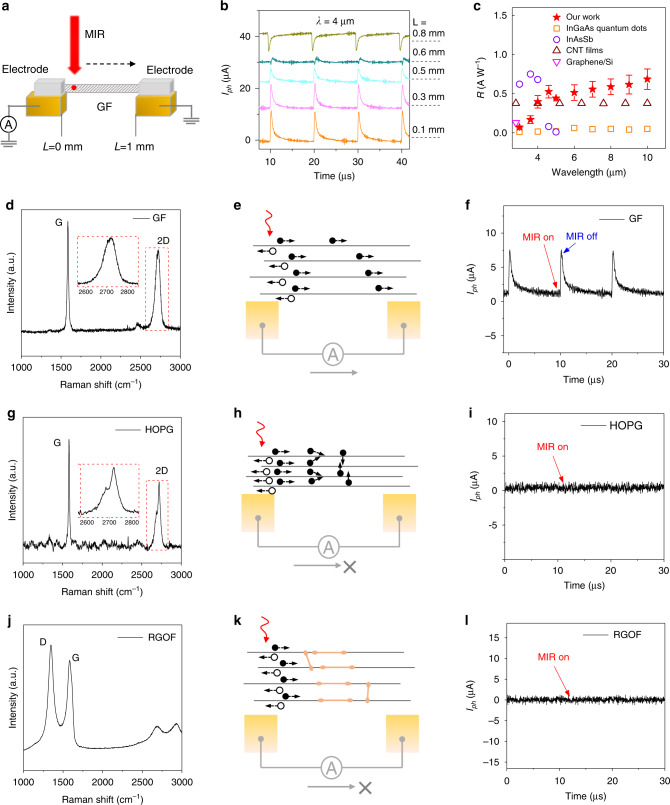
GF operation in MIR detection mode. **a** Schematic of 1 mm long GF suspended between gold electrodes illuminated by 4 μm wavelength femtosecond laser (fluence = 4.9 W cm^−2^) at constant voltage bias of 3 mV. **b** Photoresponse of GF when the laser spot moved from one electrode to the other. The position of the laser spot along the GF length is labeled next to the response signal. Photoresponse curves are shifted for clarity. **c** A comparison of responsivities of state-of-the-art MIR photodetectors with GF. Raman spectra, the corresponding MIR response, and the simplified schematics of presumed photo-carrier transport mechanisms of GF (**d**–**f**), highly oriented pyrolytic graphite (HOPG, **g**–**i**) and reduced graphene oxide fiber (RGOF, **j**–**l**), respectively. The solid circles in **e**, **h**, and **k** represent electrons, and the hollow circles represent the hole. The orange lines in **k** represent sp^3^-hybridized topologic defects.

Meters-long GFs (Fig. 1a) with ~50 μm diameter are prepared from continuously assembled wet-spun graphene oxide (GO) films using home-made twist-draw equipment^17^ (Supplementary Fig. 1), on the same scale as producing yarns or threads from cotton in the textile industry^18^. The twisting procedure imparts long-range twists (Fig. 1b), a spring-like structure (Fig. 1c) on loosely stacked graphene sheets (Fig. 1e, f) in GFs, which bring a large tensile elongation beyond 15% in the axial direction^19^ (Supplementary Fig. 1h and Movie 1), and further, GFs are woven into a natural wearable fabric (Fig. 1d). The quality of graphene sheets in GFs is verified with the high-resolution transmission electron microscope (HR-TEM) in Fig. 1g, the negligible characteristic Raman defect peak (*D* band, Fig. 2d and Supplementary Fig. 2), and the sharp reflection peak at 26.89° in X-ray diffraction (XRD) spectra (Fig. 1h). The transition from insulating to metallic behavior of conductivity with power-law dependence (Fig. 1i) suggests that GF is a clean multilayer system with minimal defect density or doping^20^. The mechanical endurance through 100 cyclical tensile tests (Fig. 1j) further demonstrates the potential of GF in durable optoelectronic devices.

### GFs in MIR detection mode

To investigate our hypothesis on GF for MIR detection, we study a 1 mm long, 50 μm diameter GF structure suspended between two terminals at room-temperature and irradiated with laser from 2 to 10 μm wavelength range. The contact resistance (*R*_C_) of the GF measured from the transfer length method (TLM) is ~1.27 Ω (Supplementary Fig. 3), and is consistent with the 4-point measurement. This value is almost an order of magnitude lower than the resistance of 1 mm long GF (12–30 Ω), and thus insignificant in the emission and detection responses of the GF. The generation of hot spot at the center of the GF and the gradual drop from the center to the electrodes along the GF while emitting MIR further supports that the maximum resistance contribution is from the GF (Supplementary Fig. 4). As shown in Fig. 2a, we record a photoresponse with a pulse width of ~0.9 μs (the rise and fall time are ~130 ns and ~4 μs, respectively) by illuminating GF with a 200-fs pulsed 4 μm MIR laser at 100 kHz repetition rate. This demonstrates that GF is capable of detecting MIR light at 0.25 MHz. Relatively higher but the opposite polarity of the photocurrent, *I*_ph_, at GF-metal interfaces and weaker response away from interfaces (Fig. 2b) at a relatively weak constant bias of 3 mV suggests that the MIR response of GF is mainly dominated by photo-thermoelectric (PTE) effect^21^. Work function differences^22^ can also induce such a built-in electric field but usually limited to nanoscale length from the interface in the bias range used in our device.

We propose that the MIR response in GFs is dominated by the photo-thermoelectric effect in the weakly coupled multilayer structure. Here, photoexcited hot electrons generated within the laser spot diffuse to GF-metal junctions by the electron temperature gradient^14,21,23,24^. We argue that unlike what is seen in nanosized devices, photo-thermoelectric effect in the GF along its length is largely influenced by interlayer hot electron transfer and scattering rather than Seebeck coefficient difference at GF-metal junction. The symmetric Raman 2D band in Fig. 2d represents the weak interlayer coupling in GFs^25^, complying randomly stacked graphene layers as displayed in Fig. 1f and the inset in Fig. 2d. Weakly coupled graphene layers can retain the mobility of single-layer graphene and increase the in-plane charge relaxation lengths^15^, and sustaining MIR response in long GFs (Fig. 2e, f). In such uncorrelated layers, hot electron relaxation length typically extends to a few micro-meters^16,24,26^. The persistence of photoresponse when the laser spot is focused at the center of the GF (*L* = 0.4 mm) indicates that the PTE effect can be extended into much longer spatial scales through multiple graphene flakes. Also, it has been reported that the hot electrons can tunnel through weakly coupled graphene layers prior to thermalization^27^ that can further increase the hot electron relaxation length.

The observed photocurrent response of GF is much broader (~900 ns) than the 200-fs incident laser pulse width. This broadening is due to the combination of resistance-capacitance (RC) time constant of electrodes, external circuit and the total cooling time of the system. The rise time of ~120 ns independent to the relative distance of the laser spot position on GF from the electrodes (Supplementary Fig. 5) supports that the photocurrent maximum is delayed due to RC of external circuit. A detailed discussion on time constant contribution of external circuit is presented in the Supplementary information. Further, trapping and de-trapping of photocarriers can increase the time of flight of carriers in GF, which can also reflect in the broadening of the photocurrent response. The extended trailing edge of ~4 μs of the photocurrent response is based on the total cooling time or the time taken for the system to reach thermal equilibrium with the surroundings. The self-suspended nature of GF and weak interlayer coupling can significantly reduce the heat dissipation to the environment and thus the lattice cooling mainly happens via thermal conductivity. Extremely slow cooling in the order of 100 μs have been recorded in carbon-based systems such as carbon nanotubes (CNTs) and graphite that are limited by the weak thermal dissipation channels and low heat capacitance^28,29^. Thus, the 4 μs trailing edge is due to above discussed factors and, therefore, we believe that the observed temporal photoresponse is a lower limit of the GF. Although, the intrinsic response of the PTE effect in GF can be much quicker (in few picoseconds), the combination of above discussed effects significantly broadens the response pulse to ~900 ns with a long trailing edge of ~4 μs in our measurement set-up.

GF exhibits the responsivity of up to 0.67 A W^−1^ in the wavelength range 2–10 μm (Fig. 2c and Supplementary Fig. 7a–h), superior to other room-temperature MIR detectors such as quantum dots^30^, black arsenic phosphorus^31^, hybrid graphene/Si^32^ or Ge photodetectors^33^, as well as photodetectors based on CNTs^34^. We propose that the comparatively higher responsivity in our device is attributed to the relaxation time and the density of hot electrons that reach GF-metal interface. At lower wavelength region, the dominant hot electron cooling mechanism is optical phonon emission. Therefore, hot electrons with energies closer to ~0.2 eV relax mostly by emitting optical phonons and thus only a limited number of hot electrons diffuse to the GF-metal interface, resulting in lower responsivity. Whereas, for the hot electron energies much lower than the optical phonon energy, the dominant cooling mechanism is via the emission of acoustic phonons, which is less efficient cooling process in graphene in mid-infrared range (2–10 μm). This enables a higher density of hot electrons to reach GF-metal interface with the increase in wavelength, and thus a gradual rise in responsivity followed by weak saturation in 6–10 μm region. We also estimated the specific detectivity (D*) and noise equivalent power (NEP) of GF in the MIR region (2–10 μm) as displayed in Supplementary Fig. 8. Minor differences in the fluence of the laser pulse at different wavelengths are depicted in error bars in the D* and NEP values. The wavelength dependency of D* is similar to the responsivity and consistent with the dominant cooling channels such as acoustic phonon emission at low photon energies. Moreover, measurements at 403 K demonstrate that the GF holds the responsivity of 0.03 A W^−1^ at comparatively higher temperatures (Supplementary Fig. 9), which offers advantage over conventional infrared detectors limited to cryogenic operation^35^.

On the other hand, the MIR photoresponse is absent in highly oriented pyrolytic graphite (HOPG, the inset in Fig. 2g, i), a strongly coupled multilayer graphene system with parabolic energy dispersion, where hot electrons quickly transfer energy to lattice due to strong electron-phonon coupling. The strongly coupled layers display splitting in the 2D Raman band (Fig. 2g), induced by orbital hybridization that can substantially increase carrier scattering and further hinders carrier transport^36^ (Fig. 2h). Besides, the quality of the graphene lattice also contributes to the fast MIR detection response. In the case of oxygen-contained defective multilayer graphene systems, such as reduced graphene oxide fibers (RGOF, Fig. 2j), charge-carriers are usually trapped by sp^3^-hybridized topologic defects^37^ (Fig. 2k), and yield no MIR response (Fig. 2l).

### GFs in MIR emission mode

**Fig. 3:**
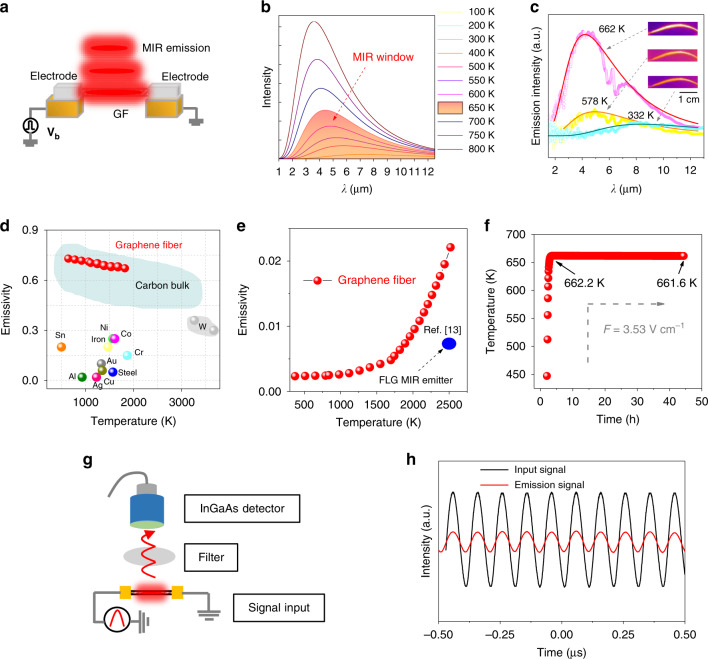
GF operation in MIR emission mode. **a** The schematic of our device. **b** The theoretical MIR window of gray-body irradiance. **c** The IR images and emission spectra (hollow dots) of GF at 332, 578, and 662 K, which match well with the gray-body radiation simulation (solid lines). The applied electric fields are 1.17 V cm^−1^, 2.35 V cm^−1^, and 3.53 V cm^−1^, respectively. The emissivity (**d**) and emission efficiency (**e**) of the GF emitter at different operation temperatures. **f**, A GF continuously emits MIR light over 40 h under a constant electric field of 3.53 V cm^−1^. The schematic (**g**) and the real-time detection (**h**) of the intensity modulation of the GF emission at 10 MHz by using a commercial InGaAs detector. The *V*_peak_ of input bias is 6 V, and the peak F is 3.53 V cm^−1^.

We investigated the MIR emission in GFs (Fig. 3a) based on gray-body radiation, where the generated thermal energy of electrically driven Joule heating is converted to MIR radiance with a broad bias-dependent spectrum^38,39^. In Fig. 3b, we plot the theoretical emission curves of a gray-body at different temperatures according to the Planck’s function: $I_\lambda \left( {\lambda ,T} \right) = \left( {\left( {2\varepsilon hc^2} \right)/\left( {\lambda ^5\left( {\exp \left( {\frac{{hc}}{{\lambda k_{\mathrm{B}}T}}} \right) - 1} \right.} \right)} \right)$, where *ε* is the emissivity, *h* is the Planck constant, *c* is the speed of light, and *K*_B_ is the Boltzmann constant. The emission spectra blue-shift to the visible light region at high temperatures and the MIR window is estimated to be below ~700 K since *λ* is mostly longer than 2 μm in this range. In the case of GF emitters, the emission spectra are easily controlled in the MIR window by directly adjusting the input electric field, *F*. Based on the emission spectra of GF in Fig. 3c, the estimated temperature is <662 K when *F* is within 3.53 V cm^−1^. The definite match of the measured emission spectra with theoretical fit indicates that the MIR emission of GF is indeed the gray-body radiation.

Apparently, GFs are effective gray-body radiators as the required *F* to operate GF emitter is at least five orders of magnitude lower than that of few-layer graphene (FLG) MIR emitters^13^. Such an improvement is related to the high *σ* (3 × 10^4^ S m^−1^, Fig. 1i), which enables GFs to consume low electrical energy and high *ε*, a factor that determines the thermal emission ability of a radiator. The strong light absorption of the multilayer graphene in GFs leads to a *ε* above 0.6 (Fig. 3d), much higher than that of single-layer graphene^40^ (SLG, ~0.02) and metals (<0.3). Such high *ε* permits GF to release high thermal energy under a low *F*. Thus, GF emitter exhibits a high thermal emission efficiency (*η*, see the Fig. 3e) of ~0.052% at 400 K, where *η* is termed as^14^: $\eta = \varepsilon \delta T^4/AP_{{\mathrm{in}}}^ \ast$. Here, *δ* is Stefan’s constant, *A* is the surface area of GF, and *P*_in_^*^ is the input electric energy per density. This value is three orders of magnitude higher than that of FLG emitters (~10^−6^)^13^. The decomposition temperature of GF approaches 800 K in the air^41^, enabling stable MIR emission of GF above room-temperature. For example, when *F* is 3.53 V cm^−1^, GF displays stable emission at ~662 K for >40 h without the loss of emissivity (Fig. 3f).

We recorded the MIR emission frequency of GF using a commercial InGaAs photodetector (Fig. 3g). As shown in Fig. 3h, we observed a synchronized MIR emission while modulating *F* up to 10 MHz. The emission speed in GF might be related to the weak interlayer coupling and the suspended structure, which minimizes vertical heat dissipation to substrate and confines most of the Joule heating^14,40^.

### MIR signal exchange between two identical GFs and demonstrations

**Fig. 4:**
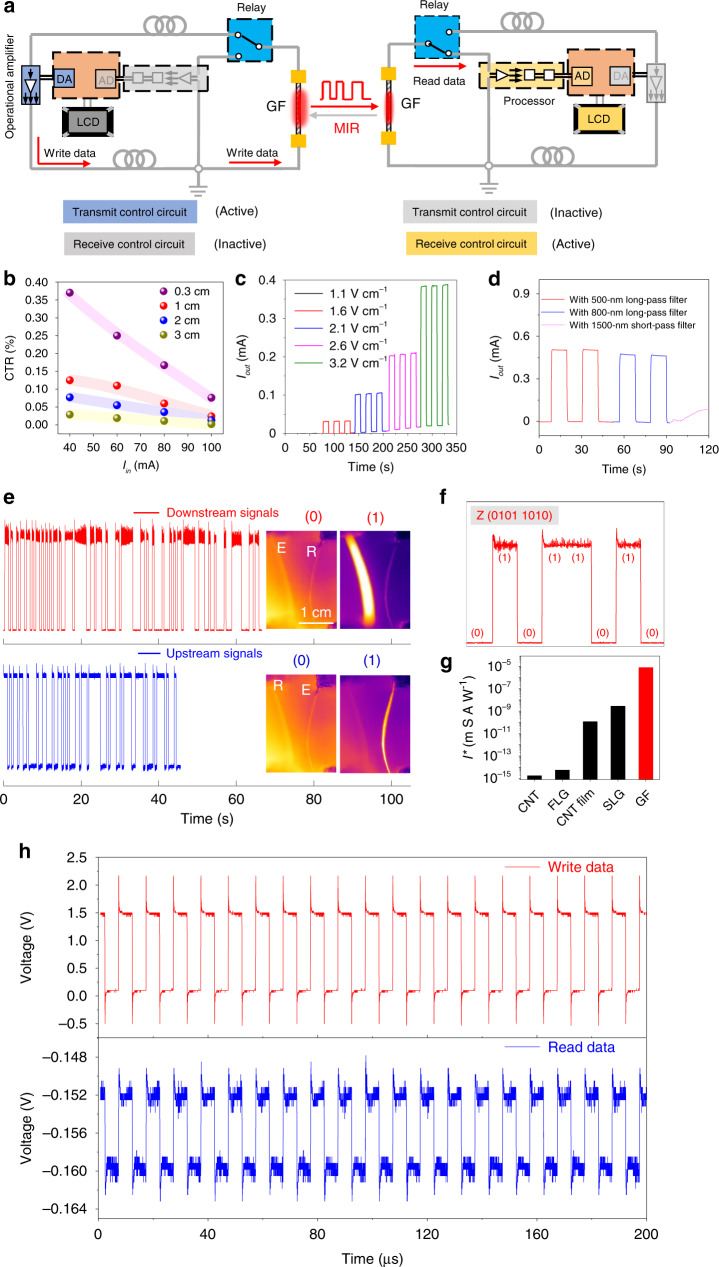
Bidirectional MIR communication system using two identical GFs. **a** Block diagram of the prototype of the bidirectional MIR communication system. Receiving and transmitting sides of the system can write/read MIR data into the system by encoding/decoding transmitting messages into binary codes. **b** The dependence of current transfer ratio (CTR) of GF on the increasing input current, *I*_in_ at various working distance (0.3–3 cm). The dependence of output current, *I*_out_, on the increasing of *F* applied to the GF emitter (**c**), and on the selection of filters (**d**) the GF–GF communication system. **e** Downstream and upstream transmissions. Details of signal processing are present in the Methods section. In IR images, “E” refers to the GF emitter and “R” refers to the GF receiver. **f** The electric signals of *E* are stably digitized in the system. Electrically driven GF transmits a signal of binary code “1”, and GF in the dormant state represents a signal of “0.” **g** The communication currents per input electrical field, I*, of state-of-the-art MIR sensors in bidirectional MIR communications. **h** MIR communication between two GFs reaches 100 KHz without the interference of signal processing modules.

Dual-functional GFs as MIR detectors and emitters, show the potential in the simplification of MIR-based optoelectronic systems. We set up a bidirectional MIR communication system (Fig. 4a and Supplementary Fig. 5) to exchange information between two identical GFs using MIR as a medium. The digital data are modulated to the on/off status of one GF, which acts as the transmitter, and the modulated MIR light is then received by the other GF and demodulated. The circuit design and working principle are explained in the Methods section. Our bidirectional communication system is switched between downstream and upstream modes by adjusting the delay and the bias voltage. The working distance is generally 0.1–3 cm, which is superior to the results in other related work^42,43^. Although the signal attenuation is inevitable (Supplementary Fig. 11), the detected photocurrent observed at the distance of 3 cm is sufficiently strong to make the bidirectional MIR communication work smoothly. As shown in Fig. 4b, the system exhibits an increasing current transfer ratio, CTR, the ratio of output current (*I*_out_, the photocurrent at the GF detector) to the input current (*I*_in_, the input current at GF emitter), with the decrease in working distance, and reaching an CTR of 0.37% at the distance of 0.3 cm. Provided that the incident MIR light from GF emitter is focused in a narrow active area of the GF receiver combined with a proper medium of transmission such as coupling with an optical fiber, high communication frequency is likely realized at much longer distances in this system.

The input electric field of GF emitter, *F*, well controls the *I*_out_ at the GF receivers (Fig. 4c), and an *F* of 3.2 V cm^−1^ generates an *I*_out_ of ~0.4 mA. Weak atmospheric absorption of light in the MIR range enables the system to achieve high transmission and also free from the interference of visible light blockers. As recorded in Fig. 4d, *I*_out_ is almost unchanged while shielding with 500 nm or 800 nm filters. The ability to switch the same GF from emission to detection eliminates the use of two sets of transmitters and receivers, resolving the problems of conventional communication systems, such as light fidelity (Li-Fi)^44^ and nonlinear conversion technique^45^. The bidirectional data transmission can modulate at 1–125 Hz (Supplementary Fig. 12). Since GF is capable of functioning at high frequencies both as a MIR emitter and detector, the communication frequency is likely limited by the RC time constants of electrodes and encoding/decoding circuitry. Thus, this communication frequency can be further improved by choosing low noise circuits that can function at high speed. As shown in Fig. 4h, MIR communication between two identical GFs reaches up to 100 kHz after removing the signal processing modules (see the supplementary Fig. 13). Both MIR downstream and upstream transmissions are highly stable (Fig. 4e, f), as demonstrated by the output of a dialog box (Supplementary Movie 2). Signal transmission in traditional communication systems are hampered by atmospheric absorption and variation in ambient pressure. In this system, the electrical signals of communication quickly stabilize at 0.45 times of initial value when the air pressure increases from 1 × 10^−5^ to 25 mbar, suggesting the ability of the GF-based MIR communication system to function at surroundings under different pressures (see the Supplementary Fig. 14).

## Discussion

The past decades have witnessed a significant development in bidirectional optical signal transmission, which made a great contribution to the monolithic integration and miniaturization of optoelectronic devices. Recently, Bao et al. proposed the bidirectional communication system between two identical perovskite-based devices^42^. However, most of this work focused on the visible and near-infrared region due to the material and performance limitations. Our system extends it to the MIR region using dual-functional and high-performance GFs. Furthermore, the MIR emission and detection properties of our GFs reflect the great potential of weakly coupled multilayer graphene systems in MIR sensors.

The GF-based bidirectional MIR communication system exhibit advances in both technology and materials. In principle, any materials that can detect MIR light are capable of bifunctionality as observed in GF, since they can emit MIR light by gray-body radiation. Since there are no practical demonstration of MIR optocoupling devices to reasonably evaluate the performance our GF system, we deduce a quality factor (I^*^, Supplementary Fig. 15), which represents *I*_out_ per input electric field, and expresses as:

1 $$I^ \ast = S\eta \varepsilon \sigma R^ \ast,$$

where *S* is the cross-sectional area with a determined radical size and R^*^ is the MIR responsivity. Judging from this relationship, we conclude that the suitable MIR sensors are required to possess a large cross-section area, high-energy conversion efficiency, high emissivity, high electrical conductivity, and high MIR responsivity. We summarize the I^*^ of state-of-the-art high-performance MIR sources and sensors, including nanocarbons such as CNT, SLG, FLG, etc. and carbon macro-materials, such as GF and CNT films. As shown in Fig. 4g, the I* of GF is 7.35 × 10^−6^ m S A W^−1^, which is at least three orders of magnitude higher than that of other carbon-based MIR sensors. Among them, SLG, FLG, and CNT are mainly limited by small *S*, while CNT film is further restricted by the low R*.

Compared to conventional semiconductor MIR emitters and receivers^46^, GFs are highly stretchable and soft. Owing to the hydrophobic nature and mechanical durability, GF withstands the strains and stresses of the textile manufacturing process and rough washing conditions (Supplementary Fig. 14a–c and Movie 3). MIR photoresponse of GF-based fabric after multiple cycles of washing remains virtually unchanged, presenting a viable path towards the wearable daily use^47^. The compatibility of GFs in wearable textile with its intrinsic merits of flexibility, room-temperature functionality, and compatibility for mass production lends well to future research towards wearable bidirectional optical communications.

## Methods

### GF fabrication

Ultra-large GO liquid crystalline dispersions in DMF were commercially purchased from GaoxiTech (www.gaoxitech.com). GO suspensions (at a concentration of 10 mg mL^−1^) were perfused into a pumping apparatus with a 2-mm-wide microfluidic channel, and then being extruded into ethyl acetate bath at a rate of 20 meters per minute. Solvents exchange and interlayer forces induce the coagulation of GO dispersions into gel belts. To guarantee the rapid drying of GO belts, we used organic solvents (DMF & EA) as the dispersive and coagulative agents. GO belts exhibit an elongation beyond 20%, and such good flexibility smooths the following twisting processing on our homemade machine. Flexible GO belts were processed into continuous graphene oxide fibers (GOFs) on a twist-draw apparatus, where a normal torque was applied to twist GO belts into a thread-like structure. After undergoing air drying, the twisted fibers were thermally treated in a vacuum oven for 2 h at 60 °C to remove residual solvents. GOFs were insulating, which were reduced into highly electrically conductive optoelectronics by chemical and thermal treatment at 3100 K.

### Characterizations

SEM images of GFs, GO belts and GO sheets were collected on a Hitachi S4800 field-emission SEM system. HR-TEM observations of the graphene sheet in GF were conducted on a JEM-2010 HR-TEM. As-prepared GFs were broken into individual graphene sheets after undergoing strong ultrasonic treatment in water. A drop of a dispersion containing individual graphene sheets was added on a copper mesh, and then were taken to HR-TEM observations after drying. Electrical conductivities were measured using the four-probe method on a Keithley 2400 multi-function source-meter. Mechanical tensile behaviors of GF were inspected on an HS-3002C at a loading rate of 10% per minute. To observe the changes in GF electrical conductivities with the increase of elongation, we combined source-meter with HS-3002C. XRD data were obtained with an X’Pert Pro (PANalytical) diffractometer employing monochromatic Cu Kα1 radiation (*λ* = 1.5406 Å) at 40 kV by adding a bundle of GFs in the field. Raman spectra were collected on a Labram HRUV spectrometer operating at 532 nm while varying the laser spot to different positions on GF.

### MIR detection measurements

To examine the MIR response, photoconductivity experiments were conducted with a GF suspended between two supporting blocks that also served as electrical contacts. Silver paste was used to glue GF on gold pads for better electrical contact. The distance between the two ends of electrical contact was 1 mm. For the convenience of comparison, all the measurement systems were placed in natural surroundings: room-temperature, relative air humidity of 40%, and illuminance of 200 Lux. The two-probe MIR response of GF was collected at various bias values in both dark and light conditions. Moreover, the light sources were produced by a femtosecond pulse MIR laser (Standard 20W model, Δ*t* = 200 fs). This laser system can emit MIR light with the wavelength from 3 to 10 μm, being equipped with integrated pulse picker, power supply, and air-water or water-water chiller. To eliminate any possible interference from lower wavelength light, we used a near-infrared (NIR) absorptive filter to purify MIR lasers in every measurement. The time-resolved response of GF was recorded by illuminating our device with a pulsed laser at low bias. By shifting the laser spots along the axial direction of GF, we collected the responsive signals at different positions. To quantitatively evaluate the MIR responsive performance of GFs, we further calculated their spectral responsivity (*R*) from the measured photocurrent (*I*_p_). *I*_p_ is obtained by subtracting the value of current under MIR irradiance from the value of dark current in *I*–*V* curves (Supplementary Fig. 5). Then *R* can be calculated as

$${\mathrm{R}} = \frac{{I_p}}{{P^ \ast A}},$$

where P* is the power density of lasers, and *A* is the illuminated area of GF.

### MIR emission measurements

GF emitter was driven by a low bias under ambient conditions with a two-terminal suspended structure. The emission spectra were collected at *Wuhan Institute of Product Quality Supervision and Inspection*, using a Jobin-Yvon iHR550 grating spectrometer, together with a cooled HgCdTe detector, with a 1–12 μm response. At such a long wavelength, MIR light could be easily absorbed by shelters, such as glass and polymer-based materials. To rule out the disturbance of MIR absorbers and achieve an accurate observation of the emission spectra, we directly collected the MIR spectra at the same surroundings like that of MIR detection measurements. In the case of gray-body irradiance, the wavelength and power of emission were entirely controlled by the input electric field (*F*). When the input *F* was lower than 3.53 V cm^−1^, GF functioned to emit a bias-dependent MIR light ranges between 2 and 10 μm. A higher *F* induces a blue-shift of emission spectra to shorter wavelengths closer to NIR and visible light region. The higher temperature produced at higher electric fields can damage our GF emitter. The MIR emission power can be calculated by the Stefan-Boltzmann law, *J* = *εσT*^4^, where *J* is the radiated power, *ε* is the emissivity, *σ* is Stefan’s constant (5.67 × 10^−8^ W m^−2^ K^−4^), and *T* is the temperature of GF. To precisely determine the real temperature (*T*) of GF at the center position, we used a double color infrared thermometer, which was able to eliminate the effects of emissivity. When *F* increases from 1.17 to 3.53 V cm^−1^, the values of corresponding *T* also increases from 372 to 578 K, and the value of MIR emission power was calculated to be at the range from 760 to 4113 W m^−2^, several orders of magnitude higher than that of previously reported MIR emitters.

### Bidirectional GF-based MIR communication system

In this system, an liquid crystal display (LCD) is used as a module to input and display digital signals. The transmit control unit is mainly composed of three parts, a digital-analog converter on a microcontroller unit (MCU), an operational amplifier, and a GF emitter. The receive control units were mainly composed of four parts, a GF receiver, a subtractor module (OP07), an amplifier module (INA114) and an analog-digital converter on MCU. To realize the bidirectional transmission of data, both the transmitting and receiving control units were connected to both the GF receiver and the emitter. To demonstrate bidirectional communication, a series of binary codes representing the message, *ZJU, do you copy?*, were transmitted through the MCU in transmit control unit, which automatically recognized the input signals as transmitted signals, and switched to the transmit mode. Further, the digital-analog converter translated the input digitals into voltage signals based on ASCII code. Then, the operational amplifier adjusted the voltage to an accurate value, ensuring the input electric field on GF emitter lies within the range of 1.13 to 3.35 V cm^−1^. Synchronous MIR light was produced from GF emitter by altering input bias. The transmitted MIR emission was received by the GF at the receiver end while varying distances in the range 0.1–3 cm away from GF emitter. Owing to a reliable MIR detecting ability, synchronous voltage signals were generated in the GF receiver with the irradiance of GF emitter. We used subtractor and amplifier modules to process weaker received signals. The subtractor module increased the spacing between light and dark voltages, and the amplifier module enhanced the overall voltage signals. Clear and smooth voltage signals were extracted from the amplifier module. Through the analog-digital converter on MCU, voltage signals were translated into digital format, which was displayed on the LCD. Thus, the downstream transmission is finished. The upstream transmission at the reverse direction is conducted by a completely symmetric process. The transmit and the receiver control units can spontaneously switch GF from emission to detection modes by using a relay.

## Supplementary information

Supplementary Information

Descriptions of Additional Supplementary Files

Supplementary Movie 1

Supplementary Movie 2

Supplementary Movie 3

## Data Availability

The data that support the findings of this study are available from the corresponding author upon reasonable request. Correspondence and requests for materials should be addressed to Y.X., Z.X., and C.G.
