# Supplementary material for: Bidirectional mid-infrared communications between two identical macroscopic graphene fibres
Source: Nat Commun. 2020 Dec 11;11:6368. doi: 10.1038/s41467-020-20033-2 (PMC7733474; doi:10.1038/s41467-020-20033-2)
Supplement: Supplementary file 1 — Supplementary Information [file 41467_2020_20033_MOESM1_ESM.pdf]

Supplementary information for

Bidirectional mid-infrared communications between two identical macroscopic graphene fibres

Bo Fang[1], Srikrishna Chanakya Bodepudi[2], Feng Tian[2,3], Xinyu Liu[2], Dan Chang[1], Sichao Du[2], Jianhang Lv[2], Jie Zhong[4], Haiming Zhu[4], Huan Hu[3], Yang Xu[2,3], Zhen Xu[1], Weiwei Gao[1], and Chao Gao[1]

[1]MOE Key Laboratory of Macromolecular Synthesis and Functionalization, Department of Polymer Science and Engineering, Zhejiang University, 38 Zheda Road, Hangzhou 310027, P. R. China.

[2]College of Microelectronic, ZJU-Hangzhou Global Scientific and Technological Innovation Centre, State Key Laboratory of Silicon Materials and Modern Optical Instruments, Zhejiang University, 38 Zheda Road, Hangzhou 310027, P.R. China.

[3]Zhejiang University/University of Illinois at Urbana-Champaign Joint Institute (ZJU-UIUC), Zhejiang University, Haining, Zhejiang 314400, P. R. China.

[4]Department of Chemistry, Zhejiang University, Hangzhou, Zhejiang 310027, P. R. China.

Bo Fang and Srikrishna Chanakya Bodepudi contributed equally to this work.

Correspondence and requests for materials should be addressed to: yangxu-isee@zju.edu.cn (Y.X.); xuzhen199@163.com (Z.X.); chaogao@zju.edu.cn (C.G.).

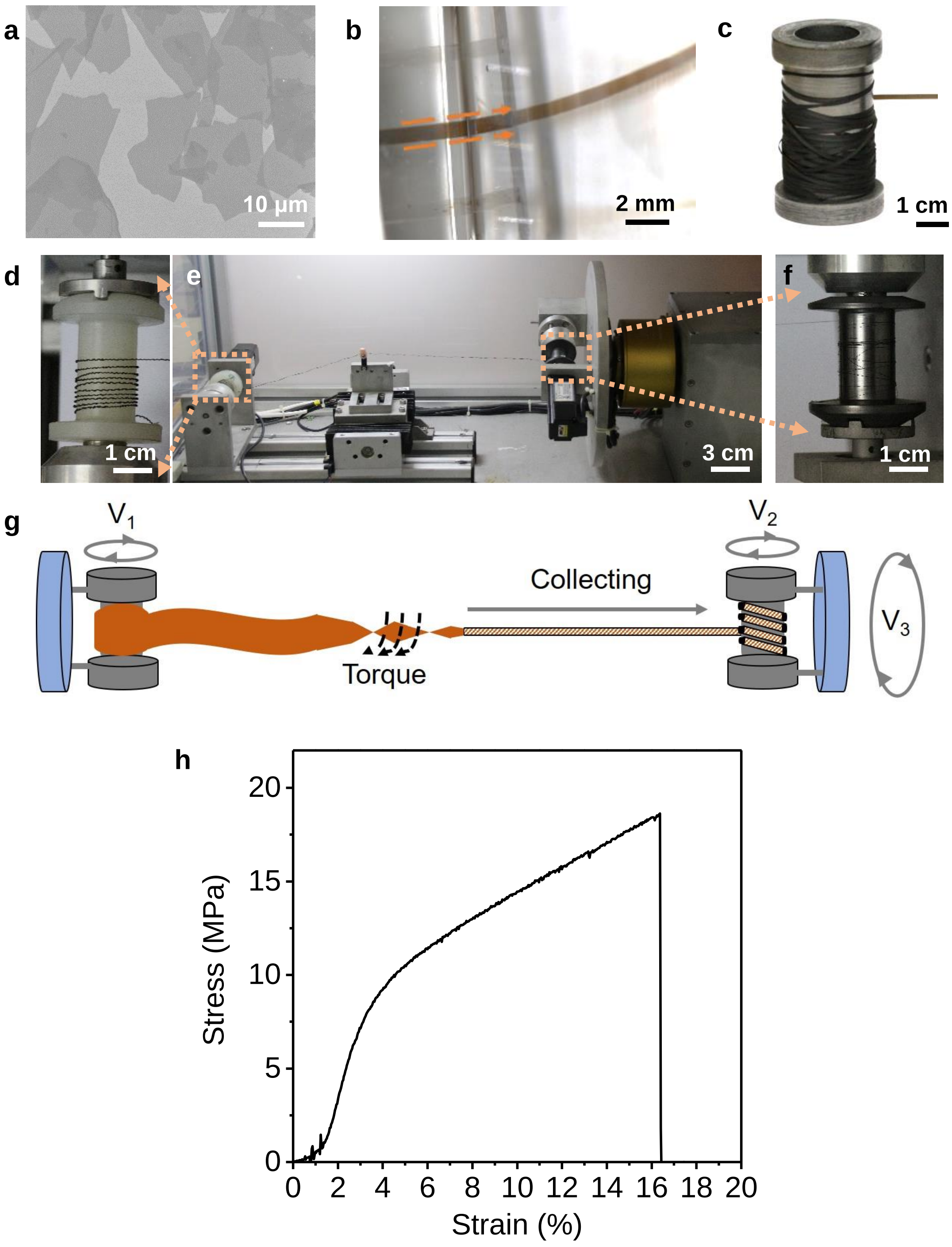

Supplementary Fig. 1 | Fabrication of continuous graphene oxide fibres (GOFs) from graphene oxide (GO) sheets. a, GO sheets with the domain size of 18 μm. **b**, The preparation of flexible GO belts. GO sheets dispersed in dimethylformamide (DMF, 10 mg mL^{-1}) pass through a microfluidic channel (a hollow gallery with a width of 2 mm and a height of 200 μm) at a rate of 20 m min^{-1}, and then being coagulated into ethyl acetate (EA) bath. Shear flow controls the orientation of GO sheets along the axial direction. The solvent exchange between DMF and EA and hydrogen-bond interactions

between interlayer GO sheets facilitates the formation of gel belts. Thus, continuous GO belts are obtained. **c**, After drying in air, the width of GO belts downsizes to 200 μm, showing favorable stretchability. **d-f,** On a homemade twisting-drawing apparatus, flexible GO belts are twisted into continuous fibres with helical microstructures on the surface. **g**, The schematic showing the operation principle of the twisting-drawing machine. On the collecting end, the rotation in two directions realizes the continuous collection and twisting of fibres. The out-plane revolution of the roller (V_3, 300 r.p.m) exerts a normal torque to the input GO belts, managing to twist the surface of GO belts from axial orientation to helical orientation. Simultaneously, the in-plane rotation of roller (V_2, 7 cm min^{-1}) is responsible for the continuous collection of graphene fibres (GFs). **h**, The typical stress-strain test of GFs.

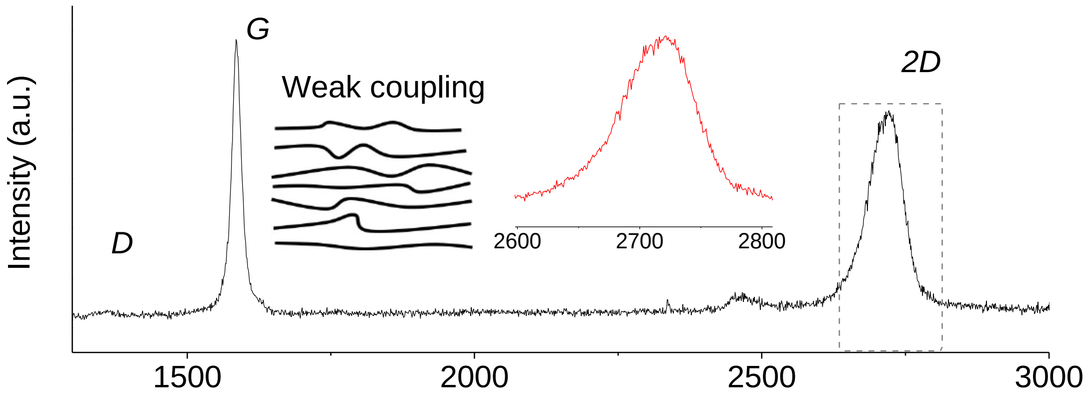

Supplementary Fig. 2 | Raman spectra of GFs after annealing at 3100 K in argon atmosphere. We randomly collected Raman spectra from 100 positions in GF. The characteristic Raman *G* band at 1571 cm^{-1} represents high-frequency E_{2g} phonon at the center of the Brillouin-zone, and *2D* band at 2699 cm^{-1} is an overtone of *D* peak. For graphene derivatives with heteroatoms, such as GO, the stronger *D* band around 1330-1350 cm^{-1} appears due to presence of large atomic defects. Negligible *D* band suggests that the heteroatomic defects in GF are mostly eliminated by high-temperature treatment. In case of graphite, where layers are AB stacked, the *2D* band at 2699 cm^{-1}consist obvious shoulder as a result of strong coupling in graphene stacks. For GFs, the symmetric *2D* band shows that there is no definite stacking order between adjacent layers.

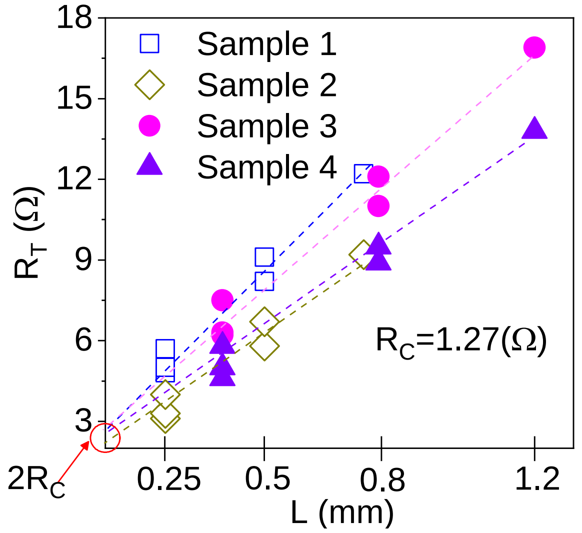

Supplementary Fig. 3 | The estimation of contact resistance from four identical samples by using the transfer length method (TLM).

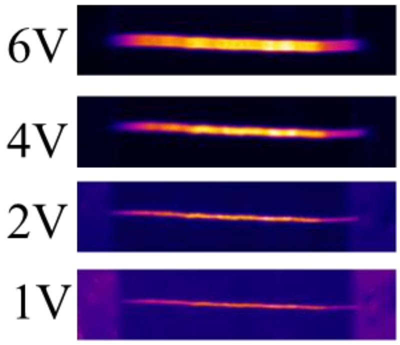

Supplementary Fig. 4 | The generation and gradual drop of hot spot from the center to the electrodes of the GF under the applied bias.

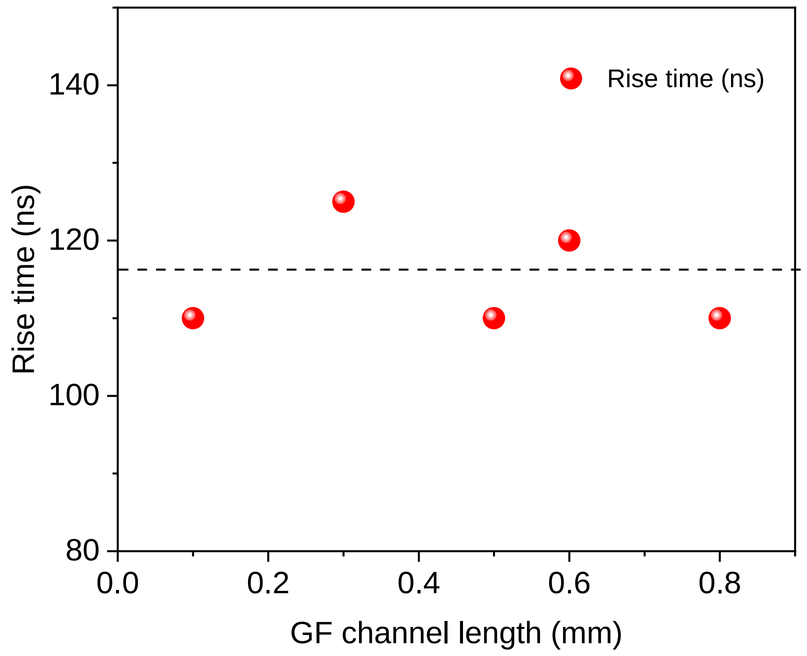

Supplementary Fig. 5 | Rise time of the photoresponse as a function of GF channel length.

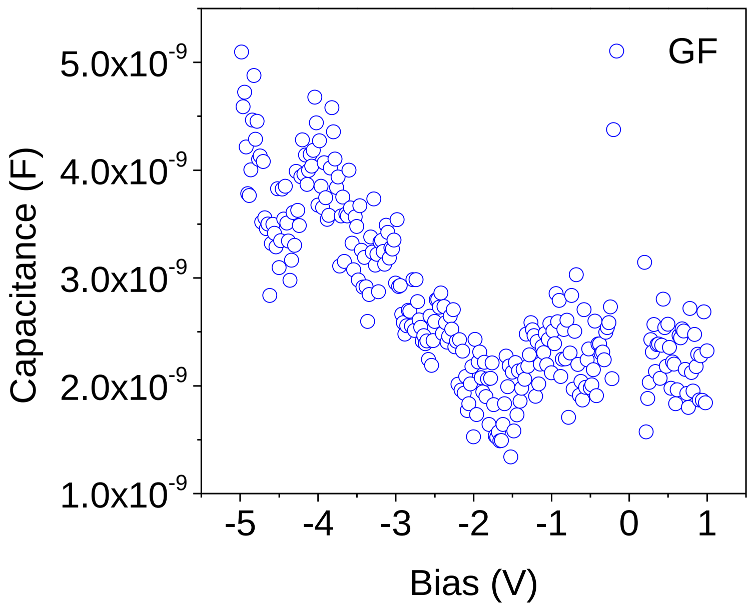

Supplementary Fig. 6 | Capacitance of GF device. The capacitance falls in the range 1.5 - 5 nF in the bias range 0 - 5 V.

To verify the *RC* contribution of electrodes, we independently measured the contact resistance and the parasitic capacitance (see the Supplementary Fig. 3 & 6), which gives

the RC time constant in 2 – 6.5 ns and a characteristic transient photocurrent maximum can be estimated as, $t_{max} = RC\, ln\left({}^{t_{tr}}/_{RC} + 1\right) \approx 12$ ns, where $t_{tr} = {}^{d}/_{v}$ is the transit time of photocarriers, d is the GF channel length, and v is the charge carrier velocity[1]. Due to weak interlayer coupling in GF, the carrier velocity is approximately two orders of magnitude lower than the Fermi velocity[2]. However, this estimated RC is still much lower than the observed value (~ 120 ns) as the above model provides the RC contribution only from the GF contacts but not from the additional circuitry such as trans-impedance amplifier (TIA) and electrical probes used in the measurement. Therefore, the major contribution of the slow rise time is presumably due to the external circuit. The rise time of the oscilloscope can also delay the photocurrent maximum which is ~ 50 ns in our system.

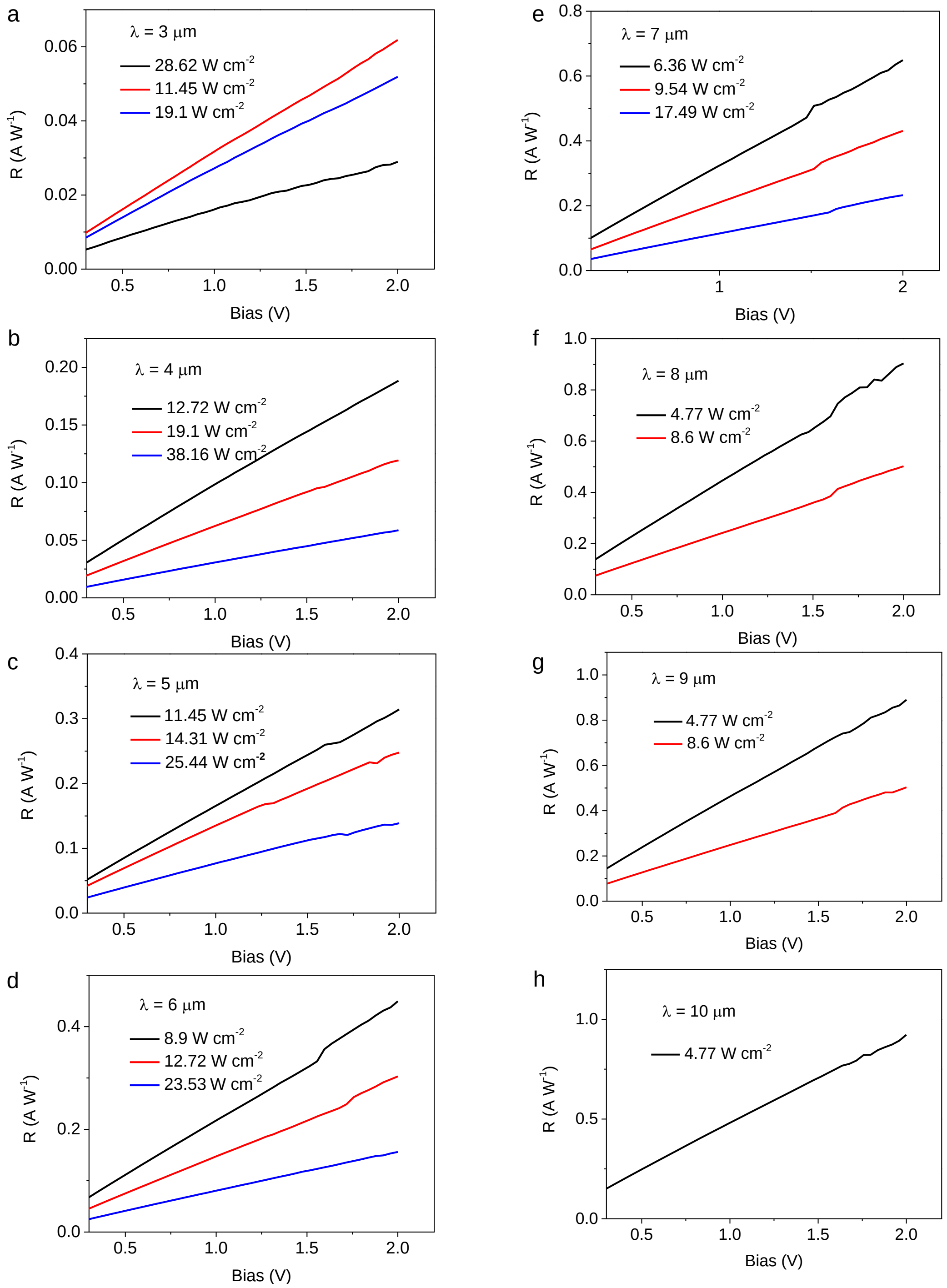

Supplementary Fig. 7 | Photoresponse of GF illuminated by the MIR laser with the wavelength of 3 µm (a), 4 µm (b), 5 µm (c), 6 µm (d), 7 µm (e), 8 µm (f), 9 µm (g) and 10 µm (h), respectively, with varying power density. The values of I_{ph} approach milliampere level, which is around three orders of magnitude higher than that of previously reported few-layer graphene (FLG) detectors. With the increase in the wavelength of lasers, we observed an increase in GF responsivity.

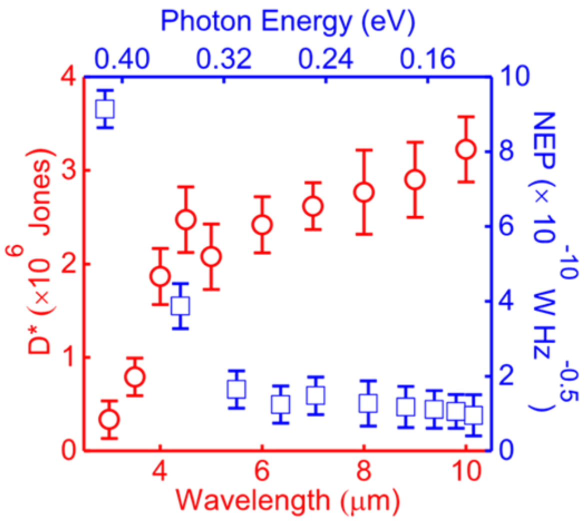

Supplementary Fig. 8 | Specific detectivity (*D*)* and noise equivalent power (NEP) of GF as a function of wavelength in the MIR region.

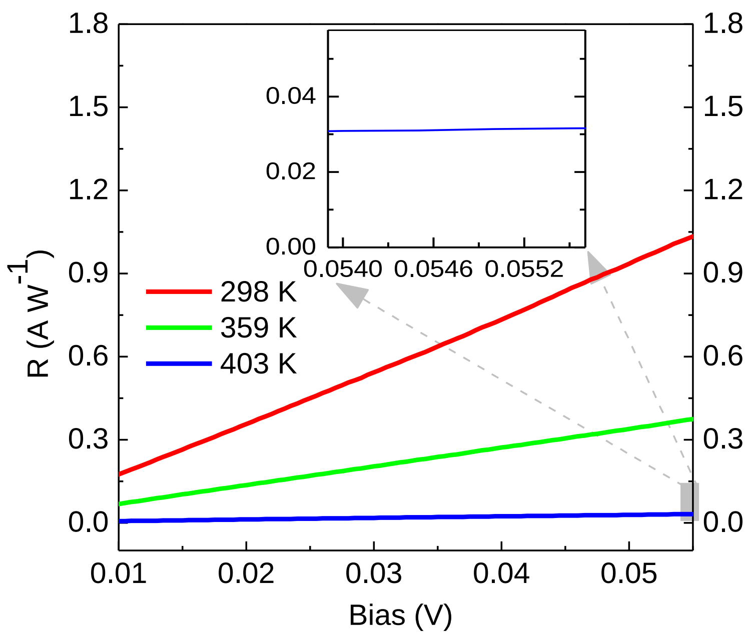

Supplementary Fig. 9 | MIR detection of GF at high-temperatures under the illumination of 2.5 μm laser. The high-temperature tests were conducted by placing the GF detector on a heating stage. To measure the accurate temperature of GF during the whole measurement process, a standard thermocouple was adjusted to the metal electrodes on the device. The heating rate was set as 5 °C min^{-1}. With the increase in temperature, the responsivity decreases gradually. MIR detection in GFs persist up to 403K where a considerable responsivity of 0.03 A W^{-1} is observed (inset enlarged figure).

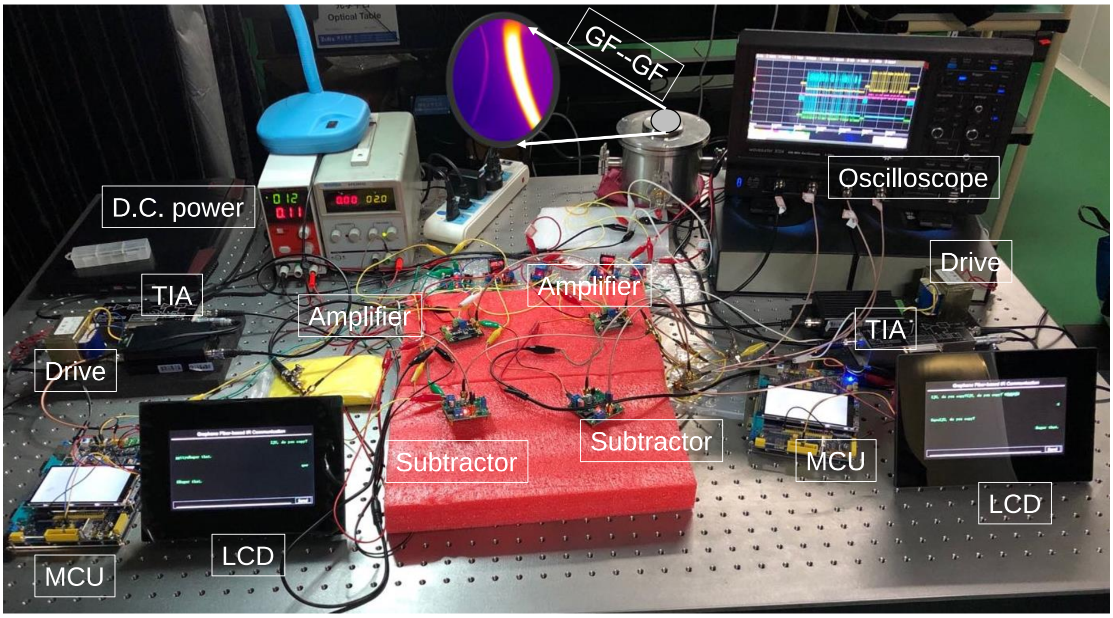

Supplementary Fig. 10 | GF-based MIR wireless communication system. The inset shows the GF emitter and detector placed in a chamber.

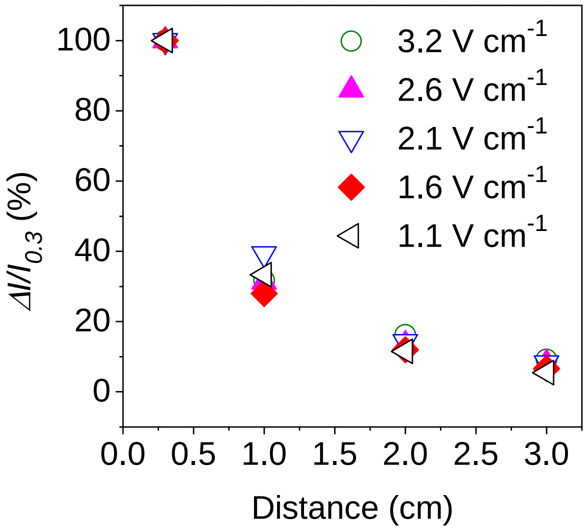

Supplementary Fig. 11 | The signal intensity-working distance relationship at different electric field (*F*). $\Delta I/I_{0.3}$ represents the ratio of changed output current at a specific working distance to the output current at the working distance of 0.3 cm.

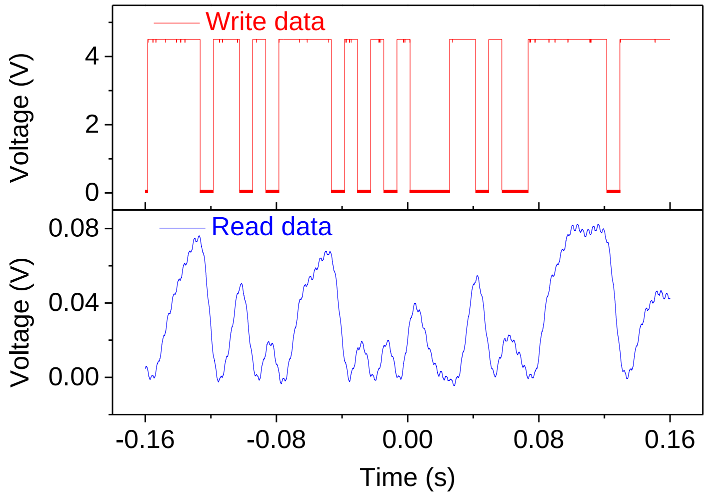

Supplementary Fig. 12 | Bidirectional MIR data transmission at 125 Hz in the system of Fig. 4a and Supplementary Video 3.

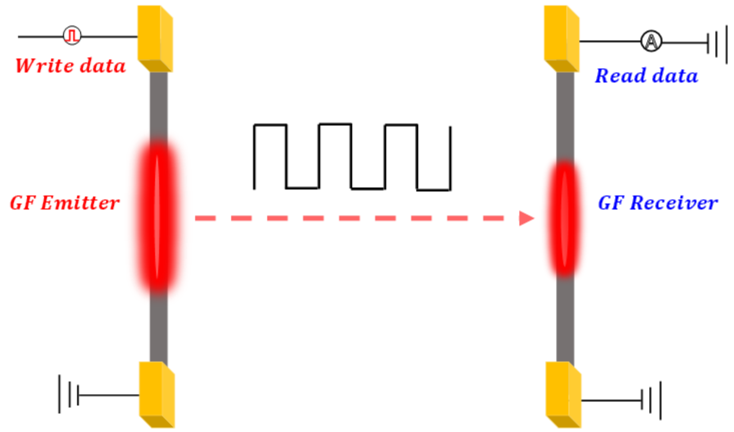

Supplementary Fig. 13 | The modified bidirectional MIR communication system without signal processing modules.

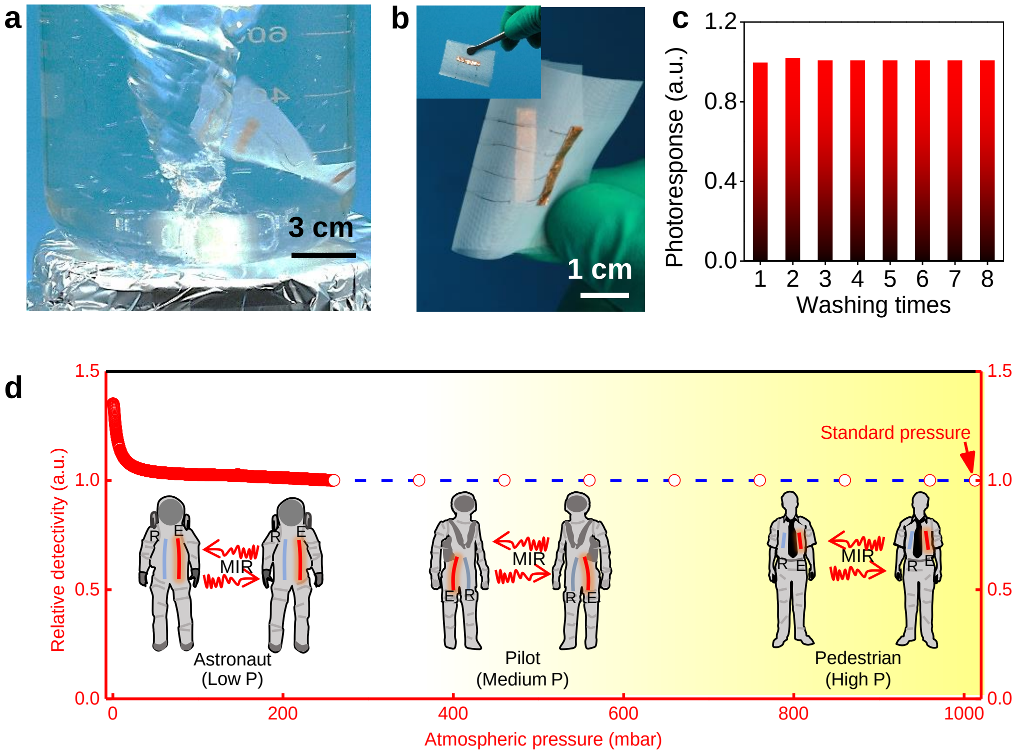

Supplementary Fig. 14 | Operation of GF-based MIR communication system at washing environment and varying atmospheric pressure. a, Washing test of an GF-based optoelectrical fabric in deionized water. The device is constructed by weaving GFs into a nylon sheet, and electrical connect is realized by soft copper electrodes. All components in this fabric, incorporating GFs, nylon sheet, and copper electrodes, are flexible and hydrophobic. **b**. GF-based optoelectrical fabric exhibits an undamaged appearance and unchanged flexibility after by being subject to vigorously stirring in water. **c**, The photoresponse of GF-based optoelectrical fabric up to eight washing cycles. Persistence of stable photoresponse after multiple washing cycles indicates its potential in practical wearable MIR communications. **d.** Due to the enhancing heat exchange between air molecules and GF optoelectronic sensor, the increasing atmospheric pressure has an influence on the communication signals of our system. However, the communication signals between the two GFs do not change largely with the increasing pressure. This makes it possible to weave the GF-based wireless MIR communication systems into the fabrics of astronauts, pilots, and pedestrians, realizing the data transmission at different operating environment.

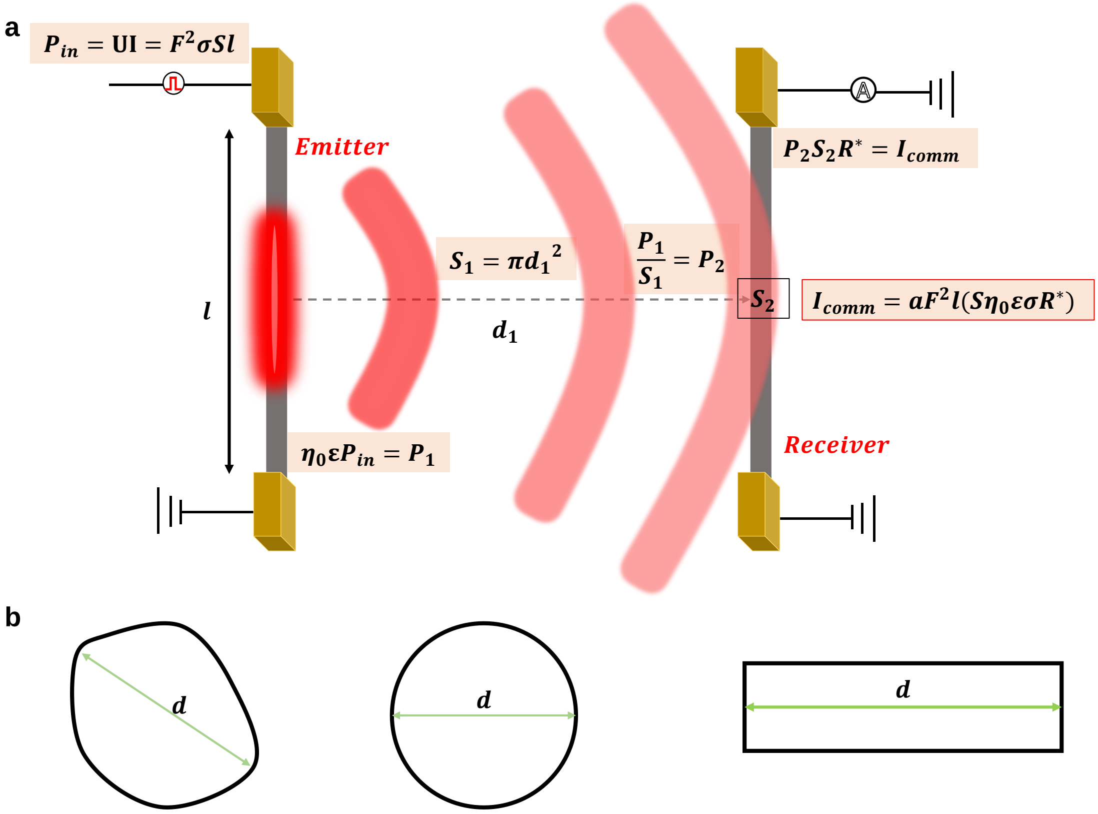

Supplementary Fig. 15 | The definition and calculation of communication current per electric field for bidirectional MIR communication. (a) The illustration of energy transfer during MIR communication between two GFs. (b) The definition of the largest-radial-size (*d*) in cross-section direction for conductors with different morphologies.

In our GF based bidirectional MIR communication system, the value of generated electrical signal in receiver, or I_{comm}, decides the quality of the whole system. Thus, we determine figure of merits for MIR communication that depends on I_{comm}.

The operation of MIR communication system is based on energy transfer. As illustrated in Supplementary Fig. 15a, the input of electrical power, $P_{in} = F^2\sigma Sl$ (Equation 1), generates Joule heating in emitter with a length of l. Thermal power, $P_1 = \eta_0 \varepsilon P_{in}$ (Equation 2), radiates from GF in a spherical form. Given that the distance between emitter and receiver is d_1, the power density of MIR irradiance at receiver is calculated as $P_2 = \frac{P_1}{S_1} = \frac{P_1}{4\pi d_1^2}$, Equation 3. Provided that the MIR responsivity of receiver is R^*, and the illumination area is S_2, the value of electrical signal in receiver is termed as $I_{comm} = P_2 S_2 R^* = aF^2 l(S\eta_0\varepsilon\sigma R^*)$, displayed in Equation 4 and 5:

$$P_{in} = \mathrm{UI} = \frac{U^2}{R} = F^2\sigma Sl \qquad (1)$$

$$\eta_0 \varepsilon P_{in} = P_1 \quad (2)$$

$$\frac{P_1}{S_1} = P_2 \quad (3)$$

$$P_2 S_2 R^* = I_{comm} \quad (4)$$

$$I_{comm} = aF^2 l(S\eta_0 \varepsilon \sigma R^*) \quad (5)$$

, where a is an arbitrary constant independent upon the inherent properties of emitters and receivers, F is the input electrical field, S is the cross-sectional area with a determined radical size (explained below), η_0 is the conversion efficiency of the energy transformed from electrical to gray-body irradiance, ε is the emissivity, and σ is the electrical conductivity.

To compare the possible values of I_{comm} in systems built by different optoelectronics, we presume that all sensors are applied a constant F and a constant l. As plotted in Supplementary Fig. 15b, considering the random morphologies of different conductors, e.g., circular metal wires and sheet-like graphene, we also confine the largest-radial-size (d) of cross-section direction to a constant value, such as 100 μm. Thus, we obtain the communication current per electrical field, I*, to determine the performance of bidirectional MIR communication, which can be expressed as:

$$I^* = S\eta_0 \varepsilon \sigma R^* \quad (6).$$

Judging from this relationship, we conclude that conductors suitable to build an optimal bidirectional MIR communication system are required to possess a large cross-section area, high energy conversion efficiency, high emissivity, high electrical conductivity, and high MIR responsivity.

Supplementary Table 1. Figure of merits of state-of-art MIR sensors

Detector	$S\ (m^2)$	η_0 (%)	ε	$\sigma\ (S\ m^{-1})$	$R^*\ (A\ W^{-1})$	I^* (m S A W^{-1}
GF	7.85×10^{-9}	0.052	0.6	3×10^{4}	0.93	7.35×10^{-6}
SLG	3×10^{-14}	0.3 [3]	0.02	1×10^{8}	1.6×10^{-2} [4]	2.7×10^{-9}
FLG	1.8×10^{-13}	1×10^{-6} [5]	0.06 [6]	$< 1 \times 10^{8}$	5×10^{-3} [7]	$< 5.4 \times 10^{-15}$
CNT	3×10^{-14}	1×10^{-6}	0.02	4.7×10^{7} [8]	1×10^{-2} [9]	1.7×10^{-15}
CNT film	1×10^{-8}	0.3 [10]	0.99 [11]	1×10^{5}	3.75×10^{-7} [12]	1.12×10^{-10}

Based on this figure of merits, we review the potential performance of state-of-art

MIR sensors, including nanocarbons (e.g., carbon nanotube (CNT), single-layer graphene (SLG), and few-layer graphene (FLG)) and carbon macro-materials (e.g., GF and CNT films). As listed in Supplementary Table 1. The I^* value of GF is 7.35 × 10^{-6} m S A W^{-1}, at least three orders of magnitude higher than that of other MIR sensors. Among of them, SLG, FLG, and CNT are mainly limited by small S. CNT film is restricted by the low R^*.

Reference

1. Kniepert, J. & Neher, D. Effect of the RC time on photocurrent transients and determination of charge carrier mobilities. *J. Appl. Phys.* **122**, 195501 (2017).
2. Richter, N. et al. Robust two-dimensional electronic properties in three-dimensional microstructures of rotationally stacked turbostratic graphene. *Phys. Rev. Appl.* **7**, 024022 (2017).
3. Kim, Y. D. et al. Bright visible light emission from graphene. *Nat. Nanotech.* **10**, 676-681 (2015).
4. Guo, Q. et al. Efficient electrical detection of mid-infrared graphene plasmons at room temperature. *Nat. Mater.* **17**, 986-992 (2018).
5. Freitag, M., Chiu, H.-Y., Steiner, M., Perebeinos V. & Avouris P. Thermal infrared emission from biased graphene. *Nat. Nanotech.* **5**, 497-501 (2010).
6. Lawton, L., Mahlmeister, N., Luxmoore, I. J. & Nash, G. R. Prospective for graphene based thermal mid-infrared light emitting devices. *AIP Adv.* **4,** 087139 (2014).
7. Yao, Y. et al. High-responsivity mid-infrared graphene detectors with antenna-enhanced photocarrier generation and collection. *Nano Lett.* **14**, 3749-3754 (2014).
8. Subramaniam, C. et al. One hundred fold increase in current carrying capacity in a carbon nanotube–copper composite. *Nat. Comm.* **4**:2202 (2013).
9. Lu, R., Christianson, C., Kirkeminde, A., Ren, S. & Wu, J. Extraordinary photocurrent harvesting at type-II heterojunction interfaces: toward high detectivity carbon nanotube infrared detectors. *Nano Lett.* **12**, 6244-6249 (2012).
10. Itkis, M. E., Yu, A. & Haddon, R. C. Single-walled carbon nanotube thin film emitter-detector integrated optoelectronic device. *Nano Lett.* **8**, 2224-2228 (2008).
11. Mizunoa, K. et al. A black body absorber from vertically aligned single-walled carbon nanotubes. *Proc. Natl. Acad. Sci.* **106**, 6044-6047 (2009).

12. Lehman, J. & Sanders, A. Very black infrared detector from vertically aligned carbon nanotubes and electric-field poling of lithium tantalite. *Nano Lett.* **10**, 3261-3266 (2010).
