# Supplementary material for: Bidirectional mid-infrared communications between two identical macroscopic graphene fibres
Source: Nat Commun. 2020 Dec 11;11:6368. doi: 10.1038/s41467-020-20033-2 (PMC7733474; doi:10.1038/s41467-020-20033-2)
Supplement: Supplementary file 2 — Descriptions of Additional Supplementary Files [file 41467_2020_20033_MOESM2_ESM.pdf]

Descriptions of Additional Supplementary Files

Supplementary Movie 1

Description: The large elongation of graphene fibre in the axial direction under the observation of optical microscope.

Supplementary Movie 2

Description: The dialogue between two graphene fibres using MIR light as the medium in the bidirectional communication system.

Supplementary Movie 3

Description: The washing test of graphene fibre-based electronic fabric in rough stirring conditions.
